# Fish Synucleins: An Update

**DOI:** 10.3390/md13116665

**Published:** 2015-10-30

**Authors:** Mattia Toni, Carla Cioni

**Affiliations:** Department of Biology and Biotechnology “Charles Darwin”, Sapienza University, Via Alfonso Borelli 50, Rome 00161, Italy; E-Mail: carla.cioni@uniroma1.it

**Keywords:** synuclein, fish, brain, zebrafish, carp

## Abstract

Synucleins (syns) are a family of proteins involved in several human neurodegenerative diseases and tumors. Since the first syn discovery in the brain of the electric ray *Torpedo californica*, members of the same family have been identified in all vertebrates and comparative studies have indicated that syn proteins are evolutionary conserved. No counterparts of syns were found in invertebrates suggesting that they are vertebrate-specific proteins. Molecular studies showed that the number of syn members varies among vertebrates. Three genes encode for α-, β- and γ-syn in mammals and birds. However, a variable number of syn genes and encoded proteins is expressed or predicted in fish depending on the species. Among biologically verified sequences, four syn genes were identified in fugu, encoding for α, β and two γ (γ1 and γ2) isoforms, whereas only three genes are expressed in zebrafish, which lacks α-syn gene. The list of “non verified” sequences is much longer and is often found in sequence databases. In this review we provide an overview of published papers and known syn sequences in agnathans and fish that are likely to impact future studies in this field. Indeed, fish models may play a key role in elucidating some of the molecular mechanisms involved in physiological and pathological functions of syn proteins.

## 1. From Fish to Human: The Discovery of the Synuclein Family

Fish played a key role in the studies in the 1980’s that led to the discovery of the first synuclein (syn) sequence. In 1980, Carlson and Kelly [1] produced a rabbit antiserum against highly purified synaptic vesicles from the electric organ of the ray *Narcine brasiliensis*. Afterwards, in 1988, Maroteaux and co-workers [2] used the same antiserum to screen a cDNA library constructed with mRNA isolated from the brain electromotor lobe of *Torpedo californica*. One gene was characterized and sequenced having a transcript of about 1.4 kb and encoding a protein with a predicted molecular weight of 14.811 Da whose expression was restricted to the central nervous system (CNS). This new protein was named “synuclein” for its predominant localization in both presynaptic terminals and neuronal nucleus. Western blot experiments performed with an antiserum against a *Torpedo* syn/B-gal fusion protein detected three protein bands at 17.5, 18.5 and 20.0 kDa in CNS homogenates from *Torpedo*. The authors concluded that syn was a protein or a series of proteins specifically expressed in the CNS and possibly involved in the coordination of nuclear and synaptic events. In the same work, Maroteaux and coworkers found a homolog to *Torpedo* syn in the rat brain cDNA library. Sequence analysis revealed about 85% identity between fish and rat syns, suggesting that the new protein is conserved among vertebrate lineages.

In 1993, a homologous protein to rat syn was discovered in the brain of patients affected by Alzheimer’s disease (AD) [3]. While studying the composition of the amyloid plaques, Ueda and co-workers [3] were able to solubilize a previously unknown peptide that was named NAC (non-aβ component of AD amyloid). Its precursor, namely NAC protein (NACP), was homologous to rat syn. In the same years, the phosphoneuroprotein 14 (PNP 14), initially identified in bovine brain, was determined to be an additional member of the syn family [4,5,6]. NACP and the human ortholog of PNP 14 were then identified as two distinct isoforms of the syn family and respectively named α- and β-syn [7]. A third variant, structurally homologous to *Torpedo* syn was added to α- and β-syn: this protein was identified in both human [8] and rat [9] and named γ-syn [10]. 

Human α-, β- and γ-syn are encoded by three distinct genes, *snca*, *sncb* and *sncg* respectively. The three genes map to different chromosomes (4q21.3-q22, 5q35 and 10q23) [10,11,12,13,14,15] and have a well preserved general organization made by five coding exons of similar size [16]. Alpha- and β-syn are predominantly expressed in the brain, particularly in neocortex, hippocampus, striatum, thalamus, and cerebellum whereas γ-syn is mainly expressed in the peripheral and the autonomic nervous system (cell bodies and axons of primary sensory, motor and sympathetic neurons) and in some tumors [8,10,17,18,19,20,21].

After the discovery of the three syns, research was primarily focused on α-syn for its involvement in several human neurodegenerative diseases known as synucleinopathies including Parkinson’s disease (PD), Parkinson’s disease dementia (PDD), dementia with Lewy bodies (DLB), multiple system atrophy (MSA), and a number of less well-characterized neuroaxonal dystrophies [22,23,24,25,26,27,28].

The universal feature of synucleinopathies is the accumulation of intracellular proteinaceous bodies containing α-syn aggregates rich in β-sheets [24]. These aggregates are called Lewy bodies in PD and PDD [29], glial cytoplasmic inclusions in MSA [30] and axonal spheroids in neuroaxonal dystrophies [31]. There is evidence indicating that molecular mechanisms involved in the pathogenesis of synucleinopathies are the misfolding of α-syn proteins into aggregates and their accumulation into intracellular bodies [29], and that neurotoxicity and cell death arise as a consequence of the dramatic α-syn accumulation [32,33].

Among synucleinopathies, the role of α-syn has been more widely investigated in PD. Single nucleotide polymorphisms in *snca* are strongly associated with the increased risk for idiopathic PD [34,35,36,37]. The *snca* missense mutation A53T was the first causal mutation identified in dominantly inherited PD [38]; other *snca* missense mutations such as A30P [39], E46K [40], H50Q [41,42], G51D [43], A53E [44] were then detected. *In vitro* experiments demonstrated that *snca* missense mutations accelerate the formation of α-syn fibrils [45]. Furthermore, *snca* duplications [46,47] and triplications [48] also lead to familiar PD suggesting that excessive amounts of normal proteins also cause PD. In addition, the formation of pathological inclusions may be promoted by α-syn post-translational modifications such as ubiquitination, nitration and phosphorylation [49,50,51].

Twenty-five years of research on syn-linked pathologies provided little advance in the discovery of physiological functions of syn proteins that still remain elusive. The collected body of evidence indicates that α-syn functions are related to its capacity to directly interact with membrane phospholipids by the highly conserved α-helical lipid-binding motif in the N-terminal region [52]. In particular, α-syn has been implicated in vesicle trafficking during neurotransmitter release [53,54], synaptic vesicle pool maintenance [55,56], synaptic plasticity [40] and learning [57]. Differently, γ-syn, first identified as breast cancer specific gene 1 (BCSG1) [8], is involved in tumor pathogenesis and correlates with adverse outcomes in breast [8,17], colon [18,19], pancreatic [20] and ovarian cancer [17]. Studies have revealed that the γ-syn overexpression leads to increased invasiveness of breast tumor cells [58] and stimulates cell proliferation [59].

Despite most research was focused on mammalian models, some advances were also made on syn expression in non-mammalian vertebrates. After Maroteaux’s pioneering work, α-syn-coding mRNAs were sequenced in representative fish, amphibians, reptiles, birds and non-primate mammals. The comparison of deduced amino acid sequences revealed that α-syns are evolutionary conserved in vertebrates [60,61,62]. Moreover, syn proteins are thought to exist only in vertebrates since no counterparts of their genes were found in the invertebrates investigated so far, *i.e.*, *Caenorhabditis elegans*, *Drosophila melanogaster* and *Ciona intestinalis* [63]. Recently, one α-syn sequence has been unexpectedly predicted from the genome of the tobacco *Nicotiana tomentosiformis* (XP_009616054.1 NCBI database). The mRNA coding sequence of the putative plant α-syn shares 100% of identity with that of human α-syn (transcript variants 1, 2, 3 and 6) and no homologous sequence is present in maize, rice and the *Arabidopsis* genome. Given the full identity between human and *Nicotiana* syn sequences, it can be assumed that the predicted tobacco syn represents a human contaminant of the plant cDNA library or a mislabeling of the sequence in NCBI. However, syn expression needs to be analyzed in tobacco cells before ruling out the putative existence of syn-like proteins in plants.

Current data on fish syns can be grouped in two sets: results from *in vivo* studies and putative nucleotide and amino acid sequences from biological databases, that were not verified in animal models. Although published papers are quite scarce, notable advancements have been made in zebrafish and carp, two ciprinids known as species models for vertebrates. Moreover, the list of putative syn sequences is continuously implemented, and we believe that an overview of available data is likely to stimulate research on the evolution of syn proteins in fish lineages. On the other hand, increasing knowledge about fish syns is helpful to develop new vertebrate models of parkinsonism for investigating relationships between syn proteins and neurodegenerative diseases.

In the present review, we provide a systematic framework for the available syn sequences of agnathans and fish. The same sequences were also compared by the maximum likelihood approach to get a first insight into the evolution of this gene family in fish. We also review the experimental studies recently performed in lamprey, fugu, zebrafish and carp.

## 2. Syn Isoforms in Agnathans and Fishes

Up to 144 nucleotide or deduced amino acid sequences of fish syns are available in the NCBI database (July 2015) from 29 species including jawless, cartilagineous and bony fishes. Most of them were obtained by RT-PCR of mRNAs extracted from blood or other tissues or predicted by computational analysis of the whole genome expression. Available sequences are summarized in Table 1. The following orders are represented: Petromyzontiformes (3 sequences), Chimaeriformes (7), Torpediniformes (1), Acipenseriformes (1), Lepisosteiformes (2), Osteoglossiformes (1), Cypriniformes (10), Characiformes (6), Siluriformes (1), Osmeriformes (1), Salmoniformes (8), Esociformes (14), Beloniformes (8), Cyprinodontiformes (16), Scorpaeniformes (1), Perciformes (43), Pleuronectiformes (7), Tetraodontiformes (11), and Coelacanthiformes (3). Twenty-five of the 29 species are ray-finned fishes and 23 of them are teleosts, belonging to the subdivisions Osteoglossomorpha (1 species), Ostarioclupeomorpha (4 species) and Euteleostei (18 species), the first two displaying more primitive morphological characters than Euteleostei. This last subdivision comprises the largest percentage of extant teleosts (94%, about 17,000 species, 346 families) [64,65]. Two more ray-finned fishes are one chondrostean and one lepisostean. The remnant four species include the lamprey, two cartilagineous fishes and one sarcopterigian. Although the picture is far from being complete, some speculations can be made on the evolutionary diversification of syns in fishes.

Three different syn sequences have been described in the lamprey *Petromyzon marinus* [66]. On the basis of the percentage of similarity with human syns the authors classified two of them as γ-syn DY (JN544525.1) and γ-syn FD (JN544526.1) whereas the third sequence was named “lamprey synuclein 3” (JN544527.1) as the percentage of identity between this protein and human syns did not allow any reliable classification.

After the discovery of *Torpedo* syn, the first study aimed at identifying the number of syn genes in the ray-finned fishes was performed in the pufferfish *Takifugu rupribes* [61]. In this tetraodontid, Yoshida and co-workers recognized four different genes that were assigned to α-(*snca*), β-(*sncb*), γ1- (*sncga* = *sncg1*) and γ2-syn (*sncgb* = *sncg2*). Each syn gene in fugu contains five coding exons showing similar size and organization to human syn genes. Interestingly, *T. rubribes* was the first species in which four genes were identified, as all vertebrates until then investigated showed three syn genes, likewise mammals [61,67].

**Table 1:** Amino acid sequences of fish synucleins including agnathans, chondrichthyans and osteichthyans available at the NCBI protein database (http://www.ncbi.nlm.nih.gov/protein). For each species the class, subclass, order and family are indicated (according to [65]). Sequences are divided in α-syn, β-syn, γ-syn and synuclein/synuclein-like (when the classification was not given). Bold numbers refer to the percentage of identity with human α-, β- and γ-syns and numbers in superscript refer to syn sequences reported in the Supplemental Data. The sequences in the same box of the table refer to identical amino acid sequences. Sequences highlighted in green, blue and red indicate X1, X2 and X3 isoforms, respectively. Dark gray, gray and light gray refer to species belonging to the subdivisions Osteoglossomorpha, Ostarioclupeomorpha and Euteleostei, respectively. Asterisks indicate the partial sequences.

Class	Subclass	Order	Family	Species	Alpha synuclein (identity with human α-syn)				Beta synuclein (identity with human β-syn)			Gamma synuclein (identity with human γ-syn)			Synuclein-like (identity with human α-syn)			
Petromyzontida		Petromyzontiformes	Petromyzontidae	*Petromyzon marinus*								**67%**^1^	**53%**^2^		**52%**^3^			
Chondrichthyes	Holocephali	Chimaeriformes	Callorhinchidae	*Callorhinchus milii*	**74%**^4,5^				**78%**^6–8^			**61%**^9^			**54%**^10^			
	Elasmobranchii	Torpediniformes	Torpedinidae	*Torpedo californica*								**59%**^11^						
Actinopterygii	Chondrostei	Acipenseriformes	Acipenseridae	*Acipenser sturio*	**94%**^12*^													
	Neopterygii	Lepisosteiformes	Lepisosteidae	*Lepisosteus oculatus*					**82%**^13^			**61%**^14^						
	Neopterygii, Division Teleostei	Osteoglossiformes	Osteoglossidae	*Scleropages formosus*								**50%**^15^						
		Cypriniformes	Cyprinidae	*Cyprinus carpio*	**99%**^16*^													
				*Danio rerio*					**71%**^17–20^			**50%**^21–23^	**52%**^24,25^					
		Characiformes	Characidae	*Astyanax mexicanus*	**55%**^26^							**35%**^27^			**60%**^28^	**53%**^29^	**28%**^30^	**52%**^31^
		Siluriformes	Siluridae	*Silurus glanis*	**99%**^32*^													
		Osmeriformes	Osmeridae	*Osmerus mordax*					**68%**^33^									
		Salmoniformes	Salmonidae	*Salmo salar*					**51%**^34,35^	**50%**^36,37^	**54%**^38^				**53%**^39,40^	**43%**^41^		
		Esociformes	Esocidae	*Esox lucius*	**52%**^42–44^	**46%**^45^	**51%**^46^	**56%**^47,48^	**56%**^49^	**55%**^50^		**54%**^51^	**58%**^52^		**53%**^53,54^	**43%**^55^		
		Beloniformes	Adrianichthyidae	*Oryzias latipes*	**61%**^56,57^	**57%**^58^			**59%**^59,60^			**49%**^61^	**50%**^62^	**55%**^63^				
		Cyprinodontiformes	Poeciliidae	*Poecilia formosa*	**62%**^64^	**46%**^65^			**61%**^66^			**55%**^67^	**46%**^68^					
				*Poecilia reticulata*	**62%**^69^	**46%**^70^			**62%**^71^			**55%**^72^	**46%**^73^					
				*Xiphophorus maculatus*	**62%**^74^				**61%**^75,76^						**56%**^77^	**55%**^78^	**50%**^79^	
		Scorpaeniformes	Anoplopomatidae	*Anoplopoma fimbria*					**59%**^80^									
		Perciformes	Cichlidae	*Astatotilapia burtoni*	**61%**^81^	**52%**^82^	**60%**^83^		**61%**^84,85^						**56%**^86^			
				*Maylandia zebra*	**61%**^87^				**61%**^88,89^			**55%**^90^	**53%**^91^		**57%**^92^			
				*Neolamprologus brichardi*	**48%**^93^	**61%**^94^			**61%**^95,96^						**57%**^97^			
				*Oreochromis niloticus*	**60%**^98^	**57%**^99^	**52%**^100^	**60%**^101^	**61%**^102^	**61%**^103^								
				*Pundamilia nyererei*	**61%**^104^	**52%**^105^	**60%**^106^		**61%**^107,108^						**57%**^109^			
			Pomacentridae	*Stegastes partitus*	**50%**^110^	**54%**^111^			**61%**^112^						**51%**^113^	**50%**^114^	**50%**^115^	**56%**^116^
			Nototheniidae	*Notothenia coriceps*	**43%**^117^	**58%**^118^			**58%**^119^									
			Sciaenidae	*Larimichthys crocea*	**61%**^120^				**62%**^121^			**53%**^122^			**57%**^123^			
		Pleuronectiformes	Cynoglossidae	*Cynoglossus semilaevis*	**54%**^124,125^				**60%**^126^			**50%**^127^	**51%**^128^		**50%**^129^	**55%**^130^		
		Tetraodontiformes	Tetraodontidae	*Takifugu rubripes*	**61%**^131,132^	**61%**^133^			**60%**^134–136^			**55%**^137,138^	**52%**^129,140^	**52%**^141^				
Sarcopterygii	Coelacanthimorpha	Coelacanthiformes	Latimeriidae	*Latimeria chalumnae*	**83%**^142^				**86%**^143^			**60%**^144^						

The presence of one additional syn gene in fugu was attributed to the whole genome duplication that occurred about 230 million years ago in the ray-finned fishes (Osteichthyes, Actinopterygii) which was followed by the subsequent loss of some duplicated genes [68,69]. On the basis of this assumption, a major variability in the number of syn isoforms can be expected in teleosts caused by events of gene duplication/loss.

A clear example of this variability is represented by zebrafish (*Danio rerio*) in which three genes were identified, *sncb* (coding for β-syn), *sncga* (γ1) and *sncgb* (γ2) [70,71,72], none of which coded for α-syn. Indeed, the zebrafish genome lacks *snca*. This absence has been explained by the putative loss of the ancestral *snca,* together with its flanking sequences, during zebrafish evolution [71]. The same studies demonstrated that zebrafish *sncb* and *sncga* give rise to single mRNA species of 1.45 kb and 2.7 kb respectively, whereas s*ncgb* is alternatively spliced to produce two transcripts encoding two isoforms with divergent C-terminals. Both *sncga* and *sncgb* contain five exons whereas *sncb* contains six exons, the first of which is noncoding. Gene structure and splice boundaries in *sncb*, *sncga*, and *sncgb* are conserved with respect to their human orthologs, providing evidence of their common origin.

At least one coding sequence for α-syn was predicted in the other fish except from *Lepisosteus ocellatus*, *Scleropages formosus*, *Osmerus mordax*, *Salmo salar* and *Anoplopoma fimbri* (Table 1). In most of them, one or more β- and γ-syn sequences were also identified.

The absence of α-syn sequences from genebank could be theoretically attributed to the absence of *snca* in respective fish genomes. If confirmed, this would suggest that the α-syn coding gene had been independently lost in distinct actinopterygian fish lineages. At the moment, however, available data cannot be interpreted in this way, as the presence of α-syn sequences was not specifically investigated in all species. Data are still scarce. Moreover, some sequences have not been classified yet as α, β- or γ-syn, being simply reported as “syn” or “syn-like” isoforms. In addition, almost all the encoded or predicted proteins were not isolated nor characterized and several accession numbers refer to gene sequences encoding the same protein.

Additional isoforms named X1, X2 or X3 are present among α-, β-, and γ-syns. The denomination “X1, X2 and X3” refers to the name reported in the NCBI database and no physiological relevance may be currently assigned to this nomenclature. X1 and X2 α-syn isoforms have been sequenced in the esocid *Esox lucius* and cichlids *Astatotilapia burtoni*, *Oreochromis niloticus and Pundamilia nyererei.* Only X1 or X2 α-syn sequences are available in *T. rubripes*, and the pomacentrid *Stegastes partitus*, respectively. Moreover, X3 α-syn isoform has been sequenced in poecilids, *Poecilia formosa* and *Poecilia reticulata*.

X1 and X2 β-syn isoforms have been sequenced in *E. lucius*, the poecilid *Xiphophorus maculates*, cichlid *Astatotilapia maculatus*, cichlids *A. burtoni*, *Neolamprologus brichardi*, *O. niloticus* and *P. nyererei*. Only X1 β-syn sequences are available in *T. rubripes*. Moreover, X1, X2 or X3 γ-syn isoforms have been sequenced in the osteoglossid *Scleropages formosus* (X2), in *E. lucius* (X1 and X2), in the adrianichthyd *Oryzias latipes* (X1, X2 and X3), in *P. formosa* and *P. reticulata* (X1), in the cichlid *Maylandia zebra* (X1 and X2), in the cynoglossid *Cynoglossus semilaevis* (X1 and X2), and in *T. rubripes* (X1).

Finally, among sequences classified as “syn” or “syn-like”, X1, X2, X3 isoforms have been sequenced in the characid *Astyanax mexicanus* (X1, X2, X3), in *X. maculatus* (X1, X2), in *S. partitus* (X1, X2) and in *C. semilaevis* (X1, X2).

Predicted fish syns are small proteins of 95–230 aa with a molecular weight (MW) ranging from 10,204 Da to 24,219 Da. Currently known α-syns correspond to proteins ranging from 102 aa (XP_010862493, *E. lucius*) to 224 aa (XP_008435281.1, *P. reticulata*) with a predicted MW of 10,204 and 23,439 Da, respectively. The 222 aa long protein XP_007562501.1 expressed in *P. formosa* showed, among fish α-syns, the highest MW of 23,458 Da. Fish β-syns are proteins with a length ranging from 115 aa (ACM08255.1 and ACI68037.1 of *S. salar*, XP_010898460.1 of *E. lucius*) to 150 aa (XP_007904439.1, *Callorhinchus milii*) with a predicted MW of 12,030 Da, 12,124 Da, 11,971 Da and 15,636 Da, respectively. Fish γ-syns range from 95 aa (XP_007234673.1, *A. mexicanus*) to 381 aa (KKX05474.1, *S. formosus*) that correspond to a predicted MW of 10,262 Da and 39,353 Da, respectively. The sequences named generally as “syn” or “syn-like” proteins consist of a number of amino acids ranging from 103 (XP_007240046.1, *A. mexicanus*) to 137 (AEO50950.1, *P. marinus*) corresponding to a MW of 10,548 and 14,244 Da, respectively.

Predicted fish syns are generally acidic proteins with an isoelectric point (pI) ranging from 4.31 to 7.75. α-syns showed a predicted pI from 4.35 (XP_010862484; *E. lucius*) to 6.20 (XP_010775984.1, *Notothenia coriiceps*); β-syns from 4.31 (ACM08255.1, *S. salar* and NP_001029018.1, *T. rubripes*) to 4.48 (XP_006631988.1, *L. ocellatus*), and γ- syns from 4.39 (NP_001029017.1, *T. rubripes*) to 7.75 (XP_007234673.1, *A. mexicanus*). The sequences generally named “syn” or “syn-like” proteins showed a predicted pI from 4.35 (XP_008297681.1, *S. partitus*) to 5.54 (XP_005943970.1, *A. burtoni*).

The comparison between fish and human syn sequences showed that fish syns share a percentage of identity from 28% to 99% with their human orthologs (Table 1). Sequence identity varies from 43% to 99% for α-syns, from 50% to 86% for β-syns and from 35% to 67% for γ-syns.

**Figure 1:**
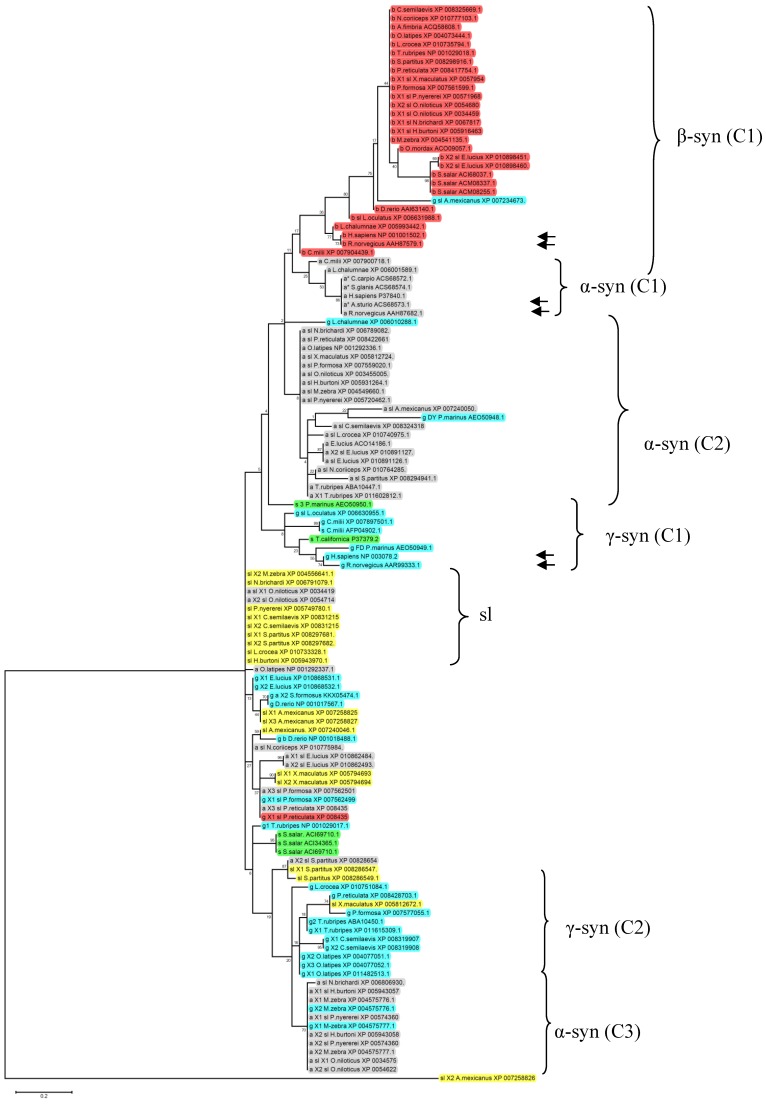
Cladogram of a ClustalX2 alignment of the ammino acid sequence of fish syns available in the NCBI database and reported in Table 1. The syn isoforms (a = α-syn; b = β-syn; g = γ-syn; s = synuclein, sl = synuclein like) with their accession numbers are shown on the right of the tree. The distance matrix employed maximum likelihood with bootstrapping (500 repetitions). Sequences highlighted in gray, orange, light blue, green and yellow refers to α-syns, β-syns, γ-syns, syn and syn-like, respectively. Arrows indicate human and rat sequences. Main sequence clusters (C) are indicated on the right of the figure.

With the aim of classifying available fish syns, we have analyzed the 144 aa sequences reported in Table 1 by Clustal W2, ProtTest 2.4 server (http://darwin.uvigo.es/software/prottest2_server.html) and MEGA 6.02 software and obtained the maximum likelihood tree represented in Figure 1. 

As shown, there is a good separation between α- and β-syns. Only five α-syn sequences clustered together with human and rat α-syns (α-syn C1). The remaining α-syns showed a lower degree of identity with the mammalian isoforms clustering in two main distant groups (α-syn C2 and C3). The α-syn C2 and C3 are composed of 19 and 9 sequences respectively. The α-syn C2 contains mainly the so-called “α-syn-like” sequences whereas α-syn C3 contains mainly X1 and X2 α-syn isoforms. The remaining 9 α-syn sequences clustered with “syn-like” proteins and γ-syns. 

The 26 fish β-syn sequences showed a good level of identity with human β-syn, grouping in the cluster β-syn C1.

Gamma-syns are widely distributed in the cladogram forming two main groups (γ-syn C1 and C2). γ-syn C1 contains fish γ-syns with the highest identity to mammalian γ-isoforms. Interestingly, γ-syns from *P. marinus* and *T. californica* were located in this cluster. The cluster γ-syn C2 contains mainly the X1, X2, and X3 γ-syn isoforms. The remaining γ-syn are distributed in the cladogram clustering mainly with X1, X2 and X3 isoform of α-syns and syn-like sequences. Moreover, nine “syn-like” sequences clustered together in the sl group.

The cladogram shows that most α-syn isoforms previously classified as α-syn-like or X1, X2 and X3 showed major affinity for γ-syn isoforms, whereas X1 and X2 β-syn have high identity percentage with mammalian β-syn.

This comparative analysis indicated that the assignment of syn sequences to α-, β- or γ-syn group is not reliable until detailed sequence information will be available. Sequence data thus needs to be implemented in more fish groups and better characterized in order to give a clear framework on how syn proteins evolved in fish lineages.

## 3. Gene Expression and Localization of Synucleins in Fish

Syn expression has been studied in lamprey (*P. marinus*), electric ray (*T. californica*), zebrafish (*D. rerio*), carp *(Cyprinus carpio*) and fugu (*T. rupribes*).

As stated above, information on syn expression first came from Maroteaux and coworker’s studies in *T. californica*. These authors identified the first syn sequence, which is now considered a γ-syn isoform [2,10]. Both gene and protein expression of *Torpedo* syn are restricted to the CNS with most abundant transcripts in the electric lobe followed by the spinal cord and the brain. In the electric organ syn proteins were only detected without their mRNA transcripts. This is consistent with the fact that syns are synthesized in neuronal cell bodies and axonally transported to nerve terminals. The subcellular localization of syn proteins was analyzed in electromotor neurons by immunohistochemistry at LM (light microscopy) and TEM (transmission electron microscopy) level and results showed that syn is present in presynaptic terminals and in cell nuclei where labeling was intense along the inner nuclear membranes and diminished toward the center of the nuclei.

The expression of the four syn isoforms was analyzed in the fugu *T. rupribes* by Western blot using specific antibodies [61]. The semiquantitative analysis showed that α-, β- and γ1-syn are expressed at similar high levels in brain homogenates, whereas γ2-syn is expressed at lower levels.

**Figure 2:**
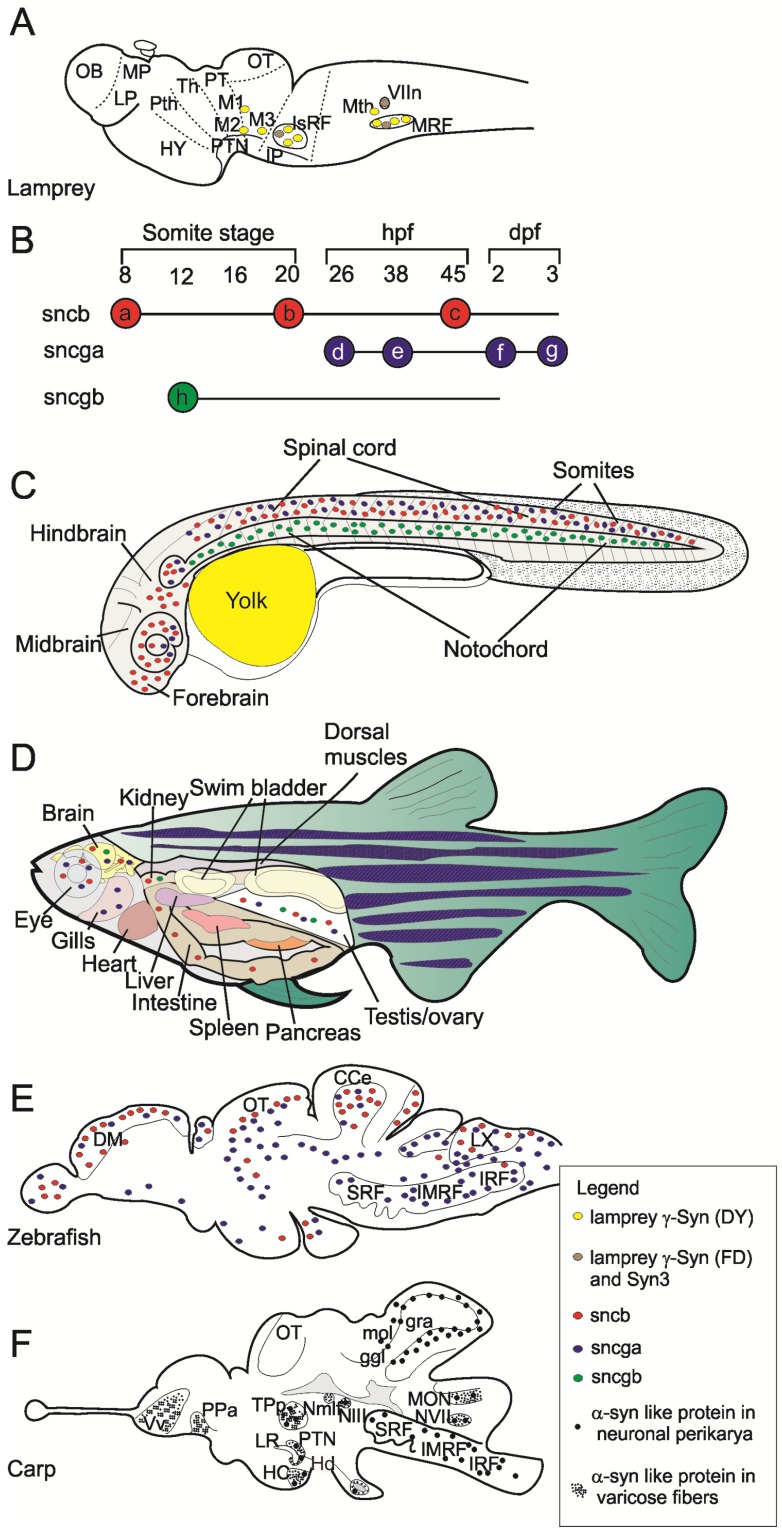
Synuclein expression in lamprey, zebrafish and carp. (**A**) Schematic representation of syn gene expression in a parasagittal section of the adult lamprey brain drawn on the base of data published by [66]; (**B**) Temporal expression pattern of syn genes in zebrafish embryo as described by [70]. The expression of *sncb* was initially detected in the trigeminal placode (a), then it expanded to ventral diencephalon, olfactory placode, ventral tegmentum, spinal cord neurons (b) and finally it was restricted to the brain and retina (c). The expression of *sncga* was initially detected in spinal cord neurons and pineal gland (d), afterwards it was detected in hindbrain neurons (e), then it was much prominent in brain and cranial ganglia (f) and finally *sncga* expression was restricted to the brain and retina (g). The expression of *sncgb* showed a different spatio-temporal distribution limited only to notochord (h); (**C**) Spatial expression pattern of syn genes in zebrafish embryo at 24 hpf (h post fertilization) as described by [71]. The *sncgb* expression is restricted to the notochord, whereas *sncb* and *sncga* are expressed in the brain and spinal cord. Red, blue and green dots indicate *sncb*, *sncga* and *sncgb* expression, respectively; (**D**): Syn genes expression pattern in the main organs of adult zebrafish on the base of results of [72]. Red, blue and green dots indicate *sncb*, *sncga* and *sncgb* expression, respectively; (**E**): Schematic representation of *sncb* and *sncga* expression pattern in a parasagittal section of the adult zebrafish brain drawn on the base of data published by [71]. Red, blue and green dots indicate *sncb*, *sncga* and *sncgb* expression, respectively. The different expression pattern of the two syn genes is well evident: *sncb* showed a most rostral expression whereas *sncga* showed a more intensive expression in posterior brain regions; (**F**): Schematic representation of the expression of α-syn-like proteins in a parasagittal section of the adult carp brain drawn on the base of data published in [73]. Large black dots indicate 3D5 immunoreactive perikarya and small black dots indicate 3D5 immunoreactive varicose fibers.

The three syns showed different levels of gene expression and regulation in the lamprey brain [66]. Higher amounts of γ-syn DY transcripts were detected in comparison to γ-syn FD and syn-3 transcripts. Moreover, the three genes were expressed to different levels in different neuronal populations. High γ-syn DY expression was detected in giant reticulospinal (RS) neurons, while γ-syn FD and syn-3 showed the highest expression levels in the facial motor nucleus (VIIn) (Figure 2A). Interestingly, the three genes were differently regulated in axotomized neurons. Indeed, mRNA levels for γ-syn DY strongly decreased in injured neurons while no changes were detected for γ-syn FD and syn-3 mRNA levels. Cellular distribution of syn proteins was analyzed by immunofluorescence using a pan-syn polyclonal antibody able to detect all three syn isoforms. Results showed that lamprey syns are located in cell soma of control RS neurons but are also associated to nuclear and plasma membranes of axotomized neurons.

More detailed information are available for zebrafish in which the expression of syn genes was analyzed in both embryos and adult animals [70,71,72]. 

Early expression of *sncb*, *sncga* and *sncgb*, showed different spatio-temporal patterns in zebrafish embryos analyzed by whole-mount *in situ* hybridization [70]. The gene *sncb* (β-syn) was first expressed from the eight-somite stage to three days post fertilization (dpf) [70]. Its expression was initially restricted to the trigeminal placode but then extended to the olfactory placode, ventral diencephalon, ventral tegmentum and spinal cord neurons by the 20-somite stage. At later stages, *sncb* expression became restricted to the brain and the retina where it was detected from 45 h post fertilization (hpf) up to 3 dpf. The expression of *sncga* (γ-synA or γ1) was not detected at early stages but initiated at 26 hpf in the nervous system beginning from spinal cord neurons and the pineal gland. It was then detected in hindbrain segmental neurons (38 hpf) becoming much more prominent in brain and cranial ganglia at two dpf. Similarly to *sncb*, the expression of *sncga* was restricted to the brain and the retina by three dpf. These data demonstrated that *sncb* and *sncga* are expressed in central and peripheral neurons irrespective of their different embryonal origin (neural plate *versus* placodes or neural crest). Very different spatio-temporal patterns were shown for *sncgb* (γ-synB or γ2) whose expression initiated earlier than *sncga*, was restricted to the notochord and was only detectable in the short time from the 12-somite stage to two dpf. Later on, *sncgb* expression diminished no more being detectable beyond 2 dpf. The spatio-temporal expression pattern of syn genes in zebrafish embryos is represented in Figure 2B,C. 

Temporal and spatial expression profiles of syn genes were also confirmed in zebrafish embryos by RT-PCR which also provided a semiquantitative evaluation of the expressional patterns [72]. Both *sncb* and *sncgb* expression were detected at early developmental stages (4 hpf) with *sncb* much more abundant than *sncgb* transcripts. Expression levels of *sncb* were intense at all developmental stages analyzed (4 hpf to 5 dpf). In contrast, levels of *sncgb* mRNAs were generally lower than those of *sncb* and further decreased by four dpf. The expression of *sncga* initiated later than *sncb* and *sncgb* (by one dpf) but its transcript level was similar to *sncb* at five dpf. The expression pattern of *sngca* was also analyzed in a transgenic stable zebrafish line. This line was obtained by microinjection into zygotes of a gene construct containing the GFP reporter gene under the control of the *sngca* promoter [72]. Expression profiles of *sngca* were obtained in transgenic embryos and larvae by whole-mount *in situ* hybridization, immunostaining, and high-resolution confocal microscopy. This analysis reported that *sncga* is expressed in both CNS and PNS of embryos and larvae. In 48 hpf embryos, GFP expression was found in retina, lens, hindbrain and spinal cord, but also in trigeminal and posterior lateral line ganglia. Similarly, at three dpf, the expression of *sngca* was detected in the diencephalon (habenula), eyes, midbrain, hindbrain, but also in trigeminal, vagal, and posterior lateral line ganglia. These results substantially confirmed the distribution of *sncga* described by Sun and Gitler [70] except for the positive staining described in the epiphysis that, according to the finest detection made by Chen *et al.* [72], is rather localized in the habenula.

Regional expression of syn genes is maintained in adult zebrafish as demonstrated by RT-PCR [72]. Thus, both *sncb* and *sncga* transcripts were particularly abundant in brain and eye, whereas *sncgb* was moderately expressed in brain and several non-nervous tissues, as testis and kidney.

The expression of syn genes was further reinvestigated in zebrafish brain and spinal cord from early embryo to adult stage by Northern blot and RNA *in situ* hybridization [71]. Northern blot analysis confirmed that both *sncb* and *sncg1* (*sncga*) are expressed in the adult CNS whereas *sncg2* (*sncgb*) transcript levels were too low to be identified. On confirming previous results, this study showed that the expression pattern of syn genes in embryos was similar to that found in adults with *sncb* and *sncg1* mainly expressed in the CNS and *sncg2* expressed in the notochord. In adult zebrafish, *sncb* and *sncg1* transcripts were both found in neurons throughout the brain and the spinal cord but their expression levels were often opposite in anteroposterior direction. Indeed, high levels of *sncb* transcripts were detected in the olfactory bulb and dorsal telencephalon where *sncg1* is expressed at low levels. Conversely, *sncg1* transcripts were abundant in the dorsal diencephalon (habenula) and hindbrain where *sncb* expression is moderate. By comparing zebrafish with lamprey it emerges that γ-syn expression in the reticular formation is conserved in the two lineages. Expression profiles of syn genes in the adult lamprey and zebrafish are depicted in Figure 2A,E.

At cellular level, *sncb* and *sncg1* were generally expressed by separate cell populations even in those brain regions in which their expression was overlapped. For example, *sncb* was mostly expressed in granules and *sncga* in Purkinje cell layer of the cerebellum. However, major catecholaminergic cell groups expressed both *sncb* and *sncg1* as demonstrated by double labeling ISH/IHC experiments. 

Further data in *Cyprinus carpio* was recently reported by our group [73]. We studied the distribution of a putative endogenous α-syn in the brain and spinal cord of the adult carp by providing the first neuroanatomical map of the α-syn distribution in the teleost CNS. The expression of α-syn was analyzed by Western blot and immunohistochemistry using a non commercial monoclonal antibody (3D5) developed against the C-terminal epitope (DMPVDPD) of human α-syn. This epitope is perfectly conserved in the carp α-syn. In brain and spinal cord homogenates, 3D5 antibody immunolabeled carp α-syn as a protein band at about 17 kDa. Western blot analysis showed that this protein is expressed to different levels in all brain regions and the spinal cord. The highest expression was detected in the midbrain tectum, followed by cerebellum and diencephalon whereas lower expression levels were found in brainstem, spinal cord and telencephalon [73].

The same 3D5 antibody was used for the immunohistochemical identification of α-syn expressing neuronal populations in the carp CNS. Positive neurons were not identified in the telencephalon but neurons expressing α-syn were localized in prethalamus, hypothalamus, synencephalon, cerebellum, brainstem and spinal cord (Figure 2F). Intracellular distribution was almost ubiquitous, since α-syn was localized in perikarya, dendrites, axons and axon terminals, except for the cell nucleus. This aspect differentiated subcellular distribution of carp α-syn from that of *Torpedo* syn, which was specifically localized to the nucleus and presynaptic terminals. This may represent a true difference between α-(carp) and γ-syn (electric ray) intracellular distribution. Moreover, since 3D5 is able to specifically recognize nuclear α-syn in mammalian neurons [74], this result suggests that α-syn is present at very low levels in the nucleus of carp neurons. Further studies on the distribution of the three syns in fishes are necessary to elucidate this point.

Our results demonstrated that carp α-syn is expressed by cholinergic ChAT immunoreactive neurons throughout the brain and the spinal cord. Differently, few subpopulations of monoaminergic neurons showed evidence of colocalization 3D5/TH or 3D5/5HT. This distribution markedly differs from expression profiles of syn genes in zebrafish [71]. However, it must be pointed out that cellular distribution of α-syn might be different from that of β and γ isoforms expressed in zebrafish. No data are yet available on cellular distribution of β- and γ-syn proteins in other fish species.

No labeling for α-syn was found in the carp striatum whereas presynaptic staining for α-syn is present in the caudate-putamen and substantia nigra of mouse [74]. As reported above, both *sncb* and *sncg1* are expressed in dopaminergic neurons of zebrafish that innervate brain structures corresponding to the teleostean striatum. Differences between zebrafish and carp need to be clarified by *in situ* hybridization and immunohistochemical studies analyzing the expression profiles of the three syns in the carp.

## 4. Functions of Fish Synucleins

Few studies addressed the issue of biochemical and physiological functions of syn proteins in fishes. Interestingly, *in vitro* experiments in fugu demonstrated that fish syns, as the mammalian isoforms, are lipid-binding proteins able to assembly themselves into filaments [61]. These biochemical properties have strong relevance in both physiological and pathological events, as they are linked to vesicle targeting of syn proteins and the formation of protein inclusions respectively. All of the four syns exhibited liposome-binding activity but α-syn had a greater affinity to liposomes compared with its human counterpart. Alpha-, γ1- and γ2-syn proteins were all able to assembly themselves into filaments at a speed eight times higher than that of mammalian isoforms. Differently, fugu β-syn failed to assembly in bulk as the human β-isoform.

Larval movements were analyzed in zebrafish treated with morpholino to inhibit *sncb* and/or *sncg1*demonstrating that β- and γ1-syn are required for normal motor activity [71].

The expression of both genes is also necessary for establishment of normal dopamine levels. Thus, it was suggested that β- and γ1-syn expressed in dopaminergic neurons of zebrafish may regulate dopamine release from which normal striatal dopamine levels and movement regulation depends. A similar regulation by dopaminergic circuits is known in mammals. Tracer studies in zebrafish sustain this suggestion having shown that dopaminergic neurons in the posterior tuberculum give rise to ascending projections to the dorsal nucleus of the ventral telencephalon, which is considered the teleostean striatum [75].

Interestingly, in the morpholino-treated zebrafish larvae, the exogenous expression of human α-syn was able to prevent the decrease in motor activity and the reduction in number of dopamine neurons.

Research in zebrafish thus suggests the functional redundancy between syn isoforms, even from different species. This supports the functional conservation of syn proteins in vertebrates and suggests that β- and γ1-syn are required for spontaneous movements and dopaminergic functions playing similar roles to those established for α-syn in humans. Nonetheless, functional properties of α-syn orthologs remain to be investigated in fishes.

## 5. Conclusions

Pathological, biochemical and genetic evidence suggest that human α-syn plays a crucial role in the pathogenesis of Parkinson’s disease (PD), dementia with Lewy bodies (DLB), Multiple System Atrophy (MSA) and tumors [29,76,77,78]. Nonetheless, the biological activity of α-syn and its precise role in neurodegeneration remain poorly understood. Fish have played a crucial role in this field since the first discovery of α-syn sequence in the electric ray *T. californica* [2]. The subsequent discovery of α- β- and γ-syns in mammals and their involvement in diseases focused further studies to mammalian models with much less interest in fish syns.

Available data highlight the species variability in the number of syn genes in fishes. Studies in fugu [61] and zebrafish [71] demonstrated the presence of four syn genes (coding α-, β-, γ1 and γ2) whereas only three genes (coding for β-, γ1 and γ2) are present in the zebrafish genome. Additional sequences for α-, β- and γ-syns have also been annotated in the NCBI database. 

Sequence data on fish syns are still limited and need to be biologically verified. However the cladogram of deduced amino acid sequences highlights the degree of identity between β-syn sequences in fish, human and rat. By contrast, only some α- and γ-syns clustered together with the mammalian counterparts. The remaining sequences were distributed in more distant clusters composed of different syn isoforms. This comparative analysis suggests that the majority of sequences first classified as X1, X2 or X3 α-syn or generically classified as “syn” or “syn-like” have a greater similarity to fish and mammalian γ-syn rather than to α- and β-syn; however exceptions are present. On the whole, these data suggest that the current syn nomenclature is still ambiguous and further studies are necessary to correctly classify fish syn sequences.

Experimental data indicated that α- (carp), β- and γ1-syn (zebrafish) are expressed in the nervous system whereas γ2 isoform (zebrafish, fugu) is moderately expressed in the brain and also in non nervous tissues. Moreover, even in the actual panorama of partial and fragmentary information, available data indicated different spatiotemporal patterns of expression for the different syn isoforms in both embryos and adult fish tissues.

Results in zebrafish and carp indicate that different syn isoforms may be expressed in separate, neurochemically different neuronal populations. In addition, intracellular syn distribution may differ with regard to nuclear localization. Indeed, nuclear localization was described for *Torpedo* (classified as γ-syn) and lamprey syns (classified as γ-syn DY, γ-syn FD and syn-3) but not in carp and zebrafish neurons.

In conclusion, data available on fish syns demonstrate a good level of sequence similarity with mammalian isoforms, the preservation of biochemical properties related to the pathogenesis of human neurodegenerative diseases and the close relationship among syns, dopamine and motor activity. Overall, this suggests that fish can be useful models for studying molecular mechanisms involved in synucleinopathies.

## Supplementary Files

Supplementary File 1

## Abbreviations

**5HT**: serotonin; **AD**: Alzheimer’s disease; **BCSG1**: breast cancer specific gene 1; **CCe**: cerebellar corpus; **ChAT**: choline acetyltransferase; **CNS**: central nervous system; **Da**:Dalton; **DLB**: dementia with Lewy bodies; **DM**: medial zone of dorsal telencephalic area; **dpf**: days post fertilization; **GFP**: green fluorescence protein; **ggl**: ganglionic layer of cerebellum; **gra**: granular layer of cerebellum **Hd**: dorsal zone of periventricular hypothalamus; **hpf**: hours post fertilization; **HY**: hypothalamus; **IMRF**: intermediate reticular formation; **IP**: interpeduncular nucleus; **IRF**: inferior reticular formation; **IsRF**: isthmic reticular formation; **LP**: lateral pallium; **LM**: light microscopy; **LR**: lateral recess of diencephalic ventricle; **LX**: vagal lobe; **M1-4**: Müller cell 1–4; **mol**: molecular layer of cerebellum **MON**: medial octavolateralis nucleus; **MP**: medial pallium; **MRF**: middle rhombencephalic reticular formation; **MSA**: multiple system atrophy; **Mth**: Mauthner cell; **MW**: molecular weight; **NAC**: non-aβ component of AD amyloid; **NACP**: NAC protein; **NIII**: oculomotor nucleus; **Nmlf**: nucleus of the medial longitudinal fascicules; **NVII**: facial motor nucleus; **OB**: olfactory bulb; **OT**: midbrain tectum; **PD**: Parkinson’s disease **PDD**: Parkinson’s disease dementia; **pI**: isoelectric point; **PNP 14**: phosphoneuroprotein 14; **PNS**: peripheral nervous system **PPa**: parvocellular preoptic nucleus, anterior part; **PT**: pretectal region; **PTh**: prethalamus (ventral thalamus); **PTN**: paratubercular nucleus; **RS**: reticulospinal; **SRF**: superior reticular formation; **syn:** synuclein; **Th**: thalamus (dorsal thalamus); **TEM**: transmission electron microscopy; **TH**: tyrosine hydroxylase. **TPp**: periventricular nucleus of posterior tuberculum; **VIIn**: facial motor nucleus.

## References

[1] Carlson S.S., Kelly R.B. (1980). An antiserum specific for cholinergic synaptic vesicles from electric organ. J. Cell Boil..

[2] Maroteaux L., Campanelli J.T., Scheller R.H. (1988). Synuclein: A neuron-specific protein localized to the nucleus and presynaptic nerve terminal. J. Neurosci. Off. J. Soc. Neurosci..

[3] Ueda K., Fukushima H., Masliah E., Xia Y., Iwai A., Yoshimoto M., Otero D.A., Kondo J., Ihara Y., Saitoh T. (1993). Molecular cloning of cDNA encoding an unrecognized component of amyloid in Alzheimer disease. Proc. Natl. Acad. Sci. USA.

[4] Tobe T., Nakajo S., Tanaka A., Mitoya A., Omata K., Nakaya K., Tomita M., Nakamura Y. (1992). Cloning and characterization of the cDNA encoding a novel brain-specific 14-kDa protein. J. Neurochem..

[5] Nakajo S., Tsukada K., Omata K., Nakamura Y., Nakaya K. (1993). A new brain-specific 14-kDa protein is a phosphoprotein. Its complete amino acid sequence and evidence for phosphorylation. Eur. J. Biochem..

[6] Shibayama-Imazu T., Okahashi I., Omata K., Nakajo S., Ochiai H., Nakai Y., Hama T., Nakamura Y., Nakaya K. (1993). Cell and tissue distribution and developmental change of neuron specific 14 kDa protein (phosphoneuroprotein 14). Brain Res..

[7] Jakes R., Spillantini M.G., Goedert M. (1994). Identification of two distinct synucleins from human brain. FEBS Lett..

[8] Ji H., Liu Y.E., Jia T., Wang M., Liu J., Xiao G., Joseph B.K., Rosen C., Shi Y.E. (1997). Identification of a breast cancer-specific gene, BCSG1, by direct differential cDNA sequencing. Cancer Res..

[9] Akopian A.N., Wood J.N. (1995). Peripheral nervous system-specific genes identified by subtractive cDNA cloning. J. Boil. Chem..

[10] Lavedan C., Leroy E., Dehejia A., Buchholtz S., Dutra A., Nussbaum R.L., Polymeropoulos M.H. (1998). Identification, localization and characterization of the human gamma-synuclein gene. Hum. Genet..

[11] Campion D., Martin C., Heilig R., Charbonnier F., Moreau V., Flaman J.M., Petit J.L., Hannequin D., Brice A., Frebourg T. (1995). The NACP/synuclein gene: Chromosomal assignment and screening for alterations in Alzheimer disease. Genomics.

[12] Chen X., de Silva H.A., Pettenati M.J., Rao P.N., St George-Hyslop P., Roses A.D., Xia Y., Horsburgh K., Ueda K., Saitoh T. (1995). The human NACP/alpha-synuclein gene: Chromosome assignment to 4q21.3-q22 and TaqI RFLP analysis. Genomics.

[13] Shibasaki Y., Baillie D.A., St Clair D., Brookes A.J. (1995). High-resolution mapping of SNCA encoding alpha-synuclein, the non-A beta component of Alzheimer’s disease amyloid precursor, to human chromosome 4q21.3→q22 by fluorescence *in situ* hybridization. Cytogenet. Cell Genet..

[14] Lavedan C., Dehejia A., Pike B., Dutra A., Leroy E., Ide S.E., Root H., Rubenstein J., Boyer R.L., Chandrasekharappa S. (1998). Contig map of the Parkinson’s disease region on 4q21-q23. DNA Res. Int. J. Rapid Publ. Rep. Genes Genomes.

[15] Spillantini M.G., Divane A., Goedert M. (1995). Assignment of human alpha-synuclein (SNCA) and beta-synuclein (SNCB) genes to chromosomes 4q21 and 5q35. Genomics.

[16] Lavedan C. (1998). The synuclein family. Genome Res..

[17] Bruening W., Giasson B.I., Klein-Szanto A.J., Lee V.M., Trojanowski J.Q., Godwin A.K. (2000). Synucleins are expressed in the majority of breast and ovarian carcinomas and in preneoplastic lesions of the ovary. Cancer.

[18] Liu C., Dong B., Lu A., Qu L., Xing X., Meng L., Wu J., Eric Shi Y., Shou C. (2010). Synuclein gamma predicts poor clinical outcome in colon cancer with normal levels of carcinoembryonic antigen. BMC Cancer.

[19] Liu C., Qu L., Dong B., Xing X., Ren T., Zeng Y., Jiang B., Meng L., Wu J., Shou C. (2012). Combined phenotype of 4 markers improves prognostic value of patients with colon cancer. Am. J. Med. Sci..

[20] Hibi T., Mori T., Fukuma M., Yamazaki K., Hashiguchi A., Yamada T., Tanabe M., Aiura K., Kawakami T., Ogiwara A. (2009). Synuclein-gamma is closely involved in perineural invasion and distant metastasis in mouse models and is a novel prognostic factor in pancreatic cancer. Clin. Cancer Res. Off. J. Am. Assoc. Cancer Res..

[21] Buchman V.L., Hunter H.J., Pinon L.G., Thompson J., Privalova E.M., Ninkina N.N., Davies A.M. (1998). Persyn, a member of the synuclein family, has a distinct pattern of expression in the developing nervous system. J. Neurosci. Off. J. Soc. Neurosci..

[22] Pfefferkorn C.M., Jiang Z., Lee J.C. (2012). Biophysics of alpha-synuclein membrane interactions. Biochim. Biophys. Acta.

[23] Dikiy I., Eliezer D. (2012). Folding and misfolding of alpha-synuclein on membranes. Biochim. Biophys. Acta.

[24] Spillantini M.G. (1999). Parkinson’s disease, dementia with Lewy bodies and multiple system atrophy are alpha-synucleinopathies. Parkinsonism Relat. Disord..

[25] Moore D.J., West A.B., Dawson V.L., Dawson T.M. (2005). Molecular pathophysiology of Parkinson’s disease. Ann. Rev. Neurosci..

[26] Luk K.C., Lee V.M. (2014). Modeling Lewy pathology propagation in Parkinson’s disease. Parkinsonism Relat. Disord..

[27] Irwin D.J., Lee V.M., Trojanowski J.Q. (2013). Parkinson’s disease dementia: Convergence of alpha-synuclein, tau and amyloid-beta pathologies. Nat. Rev. Neurosci..

[28] Norris E.H., Giasson B.I., Lee V.M. (2004). Alpha-synuclein: Normal function and role in neurodegenerative diseases. Curr. Top. Dev. Boil..

[29] Spillantini M.G., Schmidt M.L., Lee V.M., Trojanowski J.Q., Jakes R., Goedert M. (1997). Alpha-synuclein in Lewy bodies. Nature.

[30] Gai W.P., Power J.H., Blumbergs P.C., Blessing W.W. (1998). Multiple-system atrophy: A new alpha-synuclein disease?. Lancet.

[31] Newell K.L., Boyer P., Gomez-Tortosa E., Hobbs W., Hedley-Whyte E.T., Vonsattel J.P., Hyman B.T. (1999). Alpha-synuclein immunoreactivity is present in axonal swellings in neuroaxonal dystrophy and acute traumatic brain injury. J. Neuropathol. Exp. Neurol..

[32] Cookson M.R., van der Brug M. (2008). Cell systems and the toxic mechanism(s) of alpha-synuclein. Exp. Neurol..

[33] Gupta A., Dawson V.L., Dawson T.M. (2008). What causes cell death in Parkinson’s disease?. Ann. Neurol..

[34] Pals P., Lincoln S., Manning J., Heckman M., Skipper L., Hulihan M., van den Broeck M., de Pooter T., Cras P., Crook J. (2004). alpha-Synuclein promoter confers susceptibility to Parkinson’s disease. Ann. Neurol..

[35] Rajput A., Vilarino-Guell C., Rajput M.L., Ross O.A., Soto-Ortolaza A.I., Lincoln S.J., Cobb S.A., Heckman M.G., Farrer M.J., Rajput A. (2009). Alpha-synuclein polymorphisms are associated with Parkinson’s disease in a Saskatchewan population. Mov. Disord. Off. J. Mov. Disord. Soc..

[36] Pankratz N., Wilk J.B., Latourelle J.C., DeStefano A.L., Halter C., Pugh E.W., Doheny K.F., Gusella J.F., Nichols W.C., Foroud T. (2009). Genomewide association study for susceptibility genes contributing to familial Parkinson disease. Hum. Genet..

[37] Maraganore D.M., de Andrade M., Elbaz A., Farrer M.J., Ioannidis J.P., Krüger R., Rocca W.A., Schneider N.K., Lesnick T.G., Lincoln S.J. (2006). Collaborative analysis of α-synuclein gene promoter variability and Parkinson disease. J. Am. Med. Assoc..

[38] Polymeropoulos M.H., Lavedan C., Leroy E., Ide S.E., Dehejia A., Dutra A., Pike B., Root H., Rubenstein J., Boyer R. (1997). Mutation in the alpha-synuclein gene identified in families with Parkinson’s disease. Science.

[39] Kruger R., Kuhn W., Muller T., Woitalla D., Graeber M., Kosel S., Przuntek H., Epplen J.T., Schols L., Riess O. (1998). Ala30Pro mutation in the gene encoding alpha-synuclein in Parkinson’s disease. Nat. Genet..

[40] Zarranz J.J., Alegre J., Gomez-Esteban J.C., Lezcano E., Ros R., Ampuero I., Vidal L., Hoenicka J., Rodriguez O., Atares B. (2004). The new mutation, E46K, of alpha-synuclein causes Parkinson and Lewy body dementia. Ann. Neurol..

[41] Proukakis C., Dudzik C.G., Brier T., MacKay D.S., Cooper J.M., Millhauser G.L., Houlden H., Schapira A.H. (2013). A novel alpha-synuclein missense mutation in Parkinson disease. Neurology.

[42] Appel-Cresswell S., Vilarino-Guell C., Encarnacion M., Sherman H., Yu I., Shah B., Weir D., Thompson C., Szu-Tu C., Trinh J. (2013). Alpha-synuclein p.H50Q, a novel pathogenic mutation for Parkinson’s disease. Mov. Disord. Off. J. Mov. Disord. Soc..

[43] Lesage S., Anheim M., Letournel F., Bousset L., Honore A., Rozas N., Pieri L., Madiona K., Durr A., Melki R. (2013). G51D alpha-synuclein mutation causes a novel parkinsonian-pyramidal syndrome. Ann. Neurol..

[44] Pasanen P., Myllykangas L., Siitonen M., Raunio A., Kaakkola S., Lyytinen J., Tienari P.J., Poyhonen M., Paetau A. (2014). Novel alpha-synuclein mutation A53E associated with atypical multiple system atrophy and Parkinson’s disease-type pathology. Neurobiol. Aging.

[45] Conway K.A., Harper J.D., Lansbury P.T. (1998). Accelerated *in vitro* fibril formation by a mutant alpha-synuclein linked to early-onset Parkinson disease. Nat. Med..

[46] Chartier-Harlin M.C., Kachergus J., Roumier C., Mouroux V., Douay X., Lincoln S., Levecque C., Larvor L., Andrieux J., Hulihan M. (2004). Alpha-synuclein locus duplication as a cause of familial Parkinson’s disease. Lancet.

[47] Ibanez P., Bonnet A.M., Debarges B., Lohmann E., Tison F., Pollak P., Agid Y., Durr A., Brice A. (2004). Causal relation between alpha-synuclein gene duplication and familial Parkinson’s disease. Lancet.

[48] Singleton A.B., Farrer M., Johnson J., Singleton A., Hague S., Kachergus J., Hulihan M., Peuralinna T., Dutra A., Nussbaum R. (2003). alpha-Synuclein locus triplication causes Parkinson’s disease. Science.

[49] Giasson B.I., Duda J.E., Murray I.V., Chen Q., Souza J.M., Hurtig H.I., Ischiropoulos H., Trojanowski J.Q., Lee V.M. (2000). Oxidative damage linked to neurodegeneration by selective alpha-synuclein nitration in synucleinopathy lesions. Science.

[50] Tofaris G.K., Razzaq A., Ghetti B., Lilley K.S., Spillantini M.G. (2003). Ubiquitination of alpha-synuclein in Lewy bodies is a pathological event not associated with impairment of proteasome function. J. Boil. Chem..

[51] Xilouri M., Stefanis L. (2011). Autophagic pathways in Parkinson disease and related disorders. Expert Rev. Mol. Med..

[52] Iwai A., Masliah E., Yoshimoto M., Ge N., Flanagan L., de Silva H.A., Kittel A., Saitoh T. (1995). The precursor protein of non-A beta component of Alzheimer’s disease amyloid is a presynaptic protein of the central nervous system. Neuron.

[53] Chandra S., Gallardo G., Fernandez-Chacon R., Schluter O.M., Sudhof T.C. (2005). Alpha-synuclein cooperates with CSPalpha in preventing neurodegeneration. Cell.

[54] Burre J., Sharma M., Tsetsenis T., Buchman V., Etherton M.R., Sudhof T.C. (2010). Alpha-synuclein promotes SNARE-complex assembly *in vivo* and *in vitro*. Science.

[55] Abeliovich A., Schmitz Y., Farinas I., Choi-Lundberg D., Ho W.H., Castillo P.E., Shinsky N., Verdugo J.M., Armanini M., Ryan A. (2000). Mice lacking alpha-synuclein display functional deficits in the nigrostriatal dopamine system. Neuron.

[56] Murphy D.D., Rueter S.M., Trojanowski J.Q., Lee V.M. (2000). Synucleins are developmentally expressed, and alpha-synuclein regulates the size of the presynaptic vesicular pool in primary hippocampal neurons. J. Neurosci. Off. J. Soc. Neurosci..

[57] George J.M., Jin H., Woods W.S., Clayton D.F. (1995). Characterization of a novel protein regulated during the critical period for song learning in the zebra finch. Neuron.

[58] Jia T., Liu Y.E., Liu J., Shi Y.E. (1999). Stimulation of breast cancer invasion and metastasis by synuclein gamma. Cancer Res..

[59] Liu J., Spence M.J., Zhang Y.L., Jiang Y., Liu Y.E., Shi Y.E. (2000). Transcriptional suppression of synuclein gamma (SNCG) expression in human breast cancer cells by the growth inhibitory cytokine oncostatin M. Breast Cancer Res. Treat..

[60] Tiunova A.A., Anokhin K.V., Saha A.R., Schmidt O., Hanger D.P., Anderton B.H., Davies A.M., Ninkina N.N., Buchman V.L. (2000). Chicken synucleins: Cloning and expression in the developing embryo. Mech. Dev..

[61] Yoshida H., Craxton M., Jakes R., Zibaee S., Tavare R., Fraser G., Serpell L.C., Davletov B., Crowther R.A., Goedert M. (2006). Synuclein proteins of the pufferfish Fugu rubripes: Sequences and functional characterization. Biochemistry.

[62] Larsen K., Hedegaard C., Bertelsen M.F., Bendixen C. (2009). Threonine 53 in alpha-synuclein is conserved in long-living non-primate animals. Biochem. Biophys. Res. Commun..

[63] Hamilton B.A. (2004). Alpha-Synuclein A53T substitution associated with Parkinson disease also marks the divergence of Old World and New World primates. Genomics.

[64] Donaldson E.M., Evans D.H. (1993). The Physiology of Fishes.

[65] Nelson J.S. (2006). Fishes of the World.

[66] Busch D.J., Morgan J.R. (2012). Synuclein accumulation is associated with cell-specific neuronal death after spinal cord injury. J. Comp. Neurol..

[67] Yuan J., Zhao Y. (2013). Evolutionary aspects of the synuclein super-family and sub-families based on large-scale phylogenetic and group-discrimination analysis. Biochem. Biophys. Res. Commun..

[68] Amores A., Force A., Yan Y.L., Joly L., Amemiya C., Fritz A., Ho R.K., Langeland J., Prince V., Wang Y.L. (1998). Zebrafish hox clusters and vertebrate genome evolution. Science.

[69] Jaillon O., Aury J.M., Brunet F., Petit J.L., Stange-Thomann N., Mauceli E., Bouneau L., Fischer C., Ozouf-Costaz C., Bernot A. (2004). Genome duplication in the teleost fish Tetraodon nigroviridis reveals the early vertebrate proto-karyotype. Nature.

[70] Sun Z., Gitler A.D. (2008). Discovery and characterization of three novel synuclein genes in zebrafish. Dev. Dyn. Off. Publ. Am. Assoc. Anat..

[71] Milanese C., Sager J.J., Bai Q., Farrell T.C., Cannon J.R., Greenamyre J.T., Burton E.A. (2012). Hypokinesia and reduced dopamine levels in zebrafish lacking beta- and gamma1-synucleins. J. Boil. Chem..

[72] Chen Y.C., Cheng C.H., Chen G.D., Hung C.C., Yang C.H., Hwang S.P., Kawakami K., Wu B.K., Huang C.J. (2009). Recapitulation of zebrafish sncga expression pattern and labeling the habenular complex in transgenic zebrafish using green fluorescent protein reporter gene. Dev. Dyn. Off. Publ. Am. Assoc. Anat..

[73] Vaccaro R., Toni M., Casini A., Vivacqua G., Yu S., D’Este L., Cioni C. (2015). Localization of alpha-synuclein in teleost central nervous system: Immunohistochemical and Western blot evidence by 3D5 monoclonal antibody in the common carp, *Cyprinus carpio*. J. Comp. Neurol..

[74] Vivacqua G., Casini A., Vaccaro R., Fornai F., Yu S., D’Este L. (2011). Different sub-cellular localization of alpha-synuclein in the C57BL6J mouse’s central nervous system by two novel monoclonal antibodies. J. Chem. Neuroanat..

[75] Rink E., Wullimann M.F. (2001). The teleostean (zebrafish) dopaminergic system ascending to the subpallium (striatum) is located in the basal diencephalon (posterior tuberculum). Brain Res..

[76] Okazaki H., Lipkin L.E., Aronson S.M. (1961). Diffuse intracytoplasmic ganglionic inclusions (Lewy type) associated with progressive dementia and quadriparesis in flexion. J. Neuropathol. Exp. Neurol..

[77] Vilar M., Chou H.T., Luhrs T., Maji S.K., Riek-Loher D., Verel R., Manning G., Stahlberg H., Riek R. (2008). The fold of alpha-synuclein fibrils. Proc. Natl. Acad. Sci. USA.

[78] Kanaan N.M., Manfredsson F.P. (2011). Loss of functional alpha-synuclein: A toxic event in Parkinson’s disease?. J. Parkinson’s Dis..

