# Supplementary material for: Fish Synucleins: An Update
Source: Mar Drugs. 2015 Oct 30;13(11):6665–86. doi: 10.3390/md13116665 (PMC4663547; doi:10.3390/md13116665)
Supplement: Supplementary File 1 [file marinedrugs-13-06665-s001.docx]

Supplementary Materials

Numbers refer to the synuclein sequences and for each sequence the accession number, the length in amino acids, the MW and pI predicted by ProtParam at http://web.expasy.org/protparam/ are reported below. Asterisks indicate the partial sequences.

**1** [AEO50949.1; 128 aa; 13021.5 Da; 4.55 pI]; **2** [AEO50948.1; 119 aa; 11676.9 Da; 6.74 pI];
**3** [AEO50950.1; 137 aa; 14243.8 Da; 4.72 pI]; **4**,**5** [XP_007900718.1, XP_007900717.1;144 aa; 15109.8 Da; 4.56 pI]; **6**–**8** [XP_007904439.1, XP_007904438.1, AFP09332.1; 150 aa; 15636.3 Da; 4.40 pI];
**9** [XP_007897501.1; 131 aa; 13656.3 Da; 4.84 pI]; **10** [AFP04902.1; 131 aa; 13656.3 Da; 4.84 pI];
**11** [P37379.2; 143 aa; 14830.5 Da; 5.06 pI]; **12** [ACS68573.1*; 125 aa; 12644.2 Da; 5.27 pI];
**13** [XP_006631988.1; 134 aa; 14171.7 Da; 4.48 pI]; **14** [XP_006630955.1; 120 aa; 12396.8 Da;
5.03 pI]; **15** [KKX05474.1; 381 aa; 39352.7 Da; 7.70 pI]; **16** [ACS68572.1*; 125 aa; 12686.3 Da;
5.25 pI]; **17**–**20** [AAI63140.1, NP_957263.1, ACA96673.1, AAH55608.1; 127 aa; 13340.7 Da;
4.37 pI]; **21**–**23** [NP_001018488.1, AAH95672.1, ACA96674.1 (γ-1), 114 aa; 11411.7 Da; 4.68 pI]; **24**,**25** [NP_001017567.1, ACA96675.1 (γ-2); 111 aa; 11263.7 Da; 5.19 pI]; **26** [XP_007240050.1;128 aa; 13236.8 Da; 5.65 pI]; **27** [XP_007234673.1; 95 aa; 10261.7 Da; 7.75 pI]; **28** [XP_007240046.1; 103 aa; 10548.0 Da; 5.41 pI]; **29** [XP_007258825.1; 121 aa; 12387.7 Da; 4.69 pI]; **30** [XP_007258826.1; 115 ;12002.3 Da;4.87 pI]; **31** [XP_007258827.1 (X3); 114 aa; 11556.9 Da; 5.07 pI]; **32** [ACS68574.1*; 125 aa; 12686.3 Da; 5.25 pI]; **33** [ACO09057.1; 126 aa; 13353.7 Da; 4.34 pI]; **34**,**35** [ACI68037.1; NP_001135131.1; 115 aa; 12124.3 Da; 4.46 pI]; **36**,**37** [ACM08255.1, AGH92511.1; 115 aa; 12030.1 Da; 4.31 pI]; **38** [ACM08337.1; 117 aa; 12381.6 Da; 4.42 pI]; **39**,**40** [ACI34365.1-NP, 001134041.1; 109 aa; 11072.2 Da; 4.85 pI]; **41** [ACI69710.1; 130 aa; 13157.4 Da; 4.48 pI]; **42**–**44** [ACO14186.1, NP_001290689.1; XP_010891126; 159 aa; 16004.0 Da; 5.59 pI]; **45** [XP_010862484; 110 aa; 11176.3 Da; 4.35 pI]; **46** [XP_010862493; 102 aa; 10204.3 Da; 4.51 pI]; **47**,**48** [XP_010891127.1, XP_010891128.1; 130 aa; 12965.5 Da; 5.56 pI]; **49** [XP_010898451.1; 117 aa; 12228.4 Da; 4.35 pI]; **50** [XP_010898460.1; 115 aa; 11971.2 Da; 4.38 pI]; **51** [XP_010868531.1; 124 aa; 12723.0 Da; 4.49 pI]; **52** [XP_010868532.1; 109 aa; 11140.4 Da; 4.78 pI]; **53**,**54** [ACI34365.1, NP_001134041.1; 109 aa; 11072.2 Da; 4.85 pI]; **55** [ACI69710.1, 130 aa; 13157.4 Da; 4.48 pI]; **56**,**57** [NP_001292336.1, XP_004074631.1; 127 aa; 12630.0 Da; 4.67 pI]; **58** [NP_001292337.1-; 113 aa; 11492.7 Da; 4.61 pI]; **59**,**60** [XP_004073444.1; XP_011478656.1; 117 aa; 12381.6 Da; 4.38 pI]; **61** [XP_011482513.1, 152 aa; 15812.0 Da; 5.23 pI]; **62** [XP_004077051.1; 124 aa; 12353.7 Da; 4.45 pI]; **63** [XP_004077052.1; 115 aa; 11499.8 Da; 4.61 pI]; **64** [XP_007559020.1; 127 aa; 12656.0 Da; 4.54 pI]; **65** [XP_007562501.1; 222 aa; 23458.4 Da; 5.86 pI]; **66** [XP_007561599.1; 117 aa; 12417.8 Da; 4.33 pI]; **67** [XP_007577055.1; 112 aa; 11151.5 Da;5.07 pI]; **68** [XP_007562499.1; 228 aa; 24219.2 Da; 5.52 pI]; **69** [XP_008422661.1; 127 aa; 12684.0 Da; 4.56 pI]; **70** [XP_008435281.1; 224 aa; 23439.3 Da; 6.03 pI]; **71** [XP_008417754.1; 117 aa; 12403.7 Da; 4.33 pI]; **72** [XP_008428703.1; 112 aa; 11063.3 Da; 4.85 pI]; **73** [XP_008435279.1; 230 aa; 24200.1 Da; 5.73 pI]; **74** [XP_005812724.1; 127 aa; 12670.0 Da; 4.54 pI]; **75**,**76** [XP_005795466.1; XP_005795467.1; 117 aa; 12417.8 Da; 4.33 pI]; **77** [XP_005794693.1; 113 aa; 11273.5 Da; 4.50 pI]; **78** [XP_005794694.1; 110 aa ; 10959.1 Da; 4.50 pI]; **79** [XP_005812672.1; 112 aa; 11165.5 Da; 4.85 pI]; **80** [ACQ58608.1; 117 aa; 12244.5 Da; 4.36 pI]; **81** [XP_005931264; 127 aa; 12576.9 Da; 4.48 pI]; **82** [XP_005943057.1; 124 aa; 12628.9 Da; 4.40 pI]; **83** [XP_005943058.1; 115 aa; 11618.8 Da; 4.69 pI]; **84**,**85** [XP_005916463.1, XP_005916464.1; 117aa ;12371.7 Da; 4.33 pI]; **86** [XP_005943970.1; 111 aa; 11380.6 Da; 5.54 pI]; **87** [XP_004549660.1; 127 aa; 12576.9 Da; 4.48 pI]; **88**,**89** [XP_004541135.1; XP_012779429.1; 117 aa; 12371.7 Da; 4.33 pI]; **90** [XP_004575777.1; 115 aa; 11618.8 Da; 4.69 pI]; **91** [XP_004575776.1; 124 aa; 12628.9 Da; 4.40 pI]; **92** [XP_004556641.1; 113 aa; 11317.6 Da; 4.59 pI]; **93** [XP_006806930.1; 135 aa; 13994.9 Da; 4.88 pI]; **94** [XP_006789082.1; 127 aa; 12576.9 Da; 4.48 pI]; **95**,**96** [XP_006781731.1, XP_006781732.1, 117 aa; 12371.7 Da;
4.33 pI]; **97** [XP_006791079.1; 113 aa; 11317.6 Da; 4.59 pI]; **98** [XP_003455005.1; 127 aa; 12649.0 Da; 4.43 pI]; **99** [XP_003441928.1; 113 aa; 11303.5 Da; 4.56 pI]; **100** [XP_003457531.1; 124 aa; 12589.8 Da; 4.38 pI]; **101** [XP_005462259.1; 115; 11579.8 Da; 4.67 pI]; **102** [XP_003445901.1;
117 aa;12371.7 Da;4.33 pI]; **103** [XP_005468011.1; 117 aa;12371.7 Da; 4.33 pI]; **104** [XP_005720462.1; 127 aa; 12576.9 Da; 4.48 pI]; **105** [XP_005743607.1; 124 aa; 12628.9 Da; 4.40 pI]; **106** [XP_005743608.1; 115; 11618.8 Da; 4.69 pI]; **107**,**108** [XP_005719687.1, XP_005719688.1; 117 aa; 12371.7 Da; 4.33 pI]; **109** [XP_005749780.1; 113 aa; 11347.6 Da; 4.59 pI]; **110** [XP_008294941.1; 127 aa; 12822.5 Da; 4.67 pI]; **111** [XP_008286548.1; 115 aa; 11426.7 Da; 4.67 pI]; **112** [XP_008298916.1; 117 aa; 12428.7 Da; 4.33 pI]; **113** [XP_008286549.1; 111aa; 11091.5 Da; 5.02 pI]; **114** [ XP_008297681.1; 119 aa; 12126.4 Da; 4.35 pI]; **115** [XP_008286547.1; 124 aa; 12324.6 Da;
4.40 pI]; **116** [ XP_008297682.1; 113 aa; 11379.6 Da; 4.50 pI]; **117** [XP_010775984.1; 221 aa; 23445.7 Da; 6.2 pI]; **118** [XP_010764285.1; 120 aa; 12071.4 Da; 4.46 pI]; **119** [XP_010777103.1; 117 aa; 12242.5 Da; 4.38 pI]; **120** [XP_010740975.1; 127 aa; 12678.1 Da; 4.58 pI]; **121** [XP_010735794.1; 117 aa; 12446.8 Da; 4.33 pI]; **122** [XP_010751084.1; 124 aa; 12402.6 Da; 4.43 pI]; **123** [XP_010733328.1; 113 aa; 11350.5 Da; 4.50 pI]; **124**,**125** [XP_008324319.1, XP_008324318.1; 127 aa; 12579.0 Da; 4.54 pI]; **126** [XP_008325669.1; 117 aa; 12478.8 Da; 4.33 pI]; **127** [XP_008319907.1; 128 aa; 12681.9 Da ;4.45 pI]; **128** [XP_008319908.1; 119 aa; 11798.0 Da; 4.62 pI]; **129** [XP_008312152.1; 119 aa; 12167.4 Da; 4.40 pI]; **130** [XP_008312153.1; 113 aa; 11420.6 Da; 4.56 pI]; **131**,**132** [ABA10447.1, NP_001029020.1; 127 aa; 12581.0 Da; 4.52 pI]; **133** [XP_011602812.1; 127 aa; 12585.0 Da; 4.52 pI]; **134**–**136** [NP_001029018.1, XP_011609098.1, ABA10448.1; 117 aa; 12446.8 Da; 4.31 pI]; **137**,**138** [NP_001029017.1; ABA10449.1; g1, 113 aa; 11375.6 Da; 4.39 pI]; **139**,**140** [ABA10450.1, NP_001029019.1 (g-2), 124 aa; 12461.8 Da; 4.54 pI]; **141** [XP_011615309.1; 124 aa ; 12489.8 Da; 4.54 pI]; **142** [XP_006001589.1; 144 aa; 15140.0 Da; 4.83 pI]; **143** [XP_005993442.1; 133 aa; 14006.6 Da; 4.43 pI]; **144** [XP_006010288.1; 121 aa; 12399.8 Da; 4.94 pI].

© 2015 by the authors; licensee MDPI, Basel, Switzerland. This article is an open access article distributed under the terms and conditions of the Creative Commons Attribution license (http://creativecommons.org/licenses/by/4.0/).
